# Phylogenetic Analysis of a Microbialite-Forming Microbial Mat from a Hypersaline Lake of the Kiritimati Atoll, Central Pacific

**DOI:** 10.1371/journal.pone.0066662

**Published:** 2013-06-10

**Authors:** Dominik Schneider, Gernot Arp, Andreas Reimer, Joachim Reitner, Rolf Daniel

**Affiliations:** 1 Department of Genomic and Applied Microbiology and Göttingen Genomics Laboratory, Institute of Microbiology and Genetics, Georg-August University Göttingen, Göttingen, Germany; 2 Geoscience Centre, Georg-August University Göttingen, Göttingen, Germany; Catalan Institute for Water Research (ICRA), Spain

## Abstract

On the Kiritimati atoll, several lakes exhibit microbial mat-formation under different hydrochemical conditions. Some of these lakes trigger microbialite formation such as Lake 21, which is an evaporitic, hypersaline lake (salinity of approximately 170‰). Lake 21 is completely covered with a thick multilayered microbial mat. This mat is associated with the formation of decimeter-thick highly porous microbialites, which are composed of aragonite and gypsum crystals. We assessed the bacterial and archaeal community composition and its alteration along the vertical stratification by large-scale analysis of 16S rRNA gene sequences of the nine different mat layers. The surface layers are dominated by aerobic, phototrophic, and halotolerant microbes. The bacterial community of these layers harbored *Cyanobacteria* (*Halothece* cluster), which were accompanied with known phototrophic members of the *Bacteroidetes* and *Alphaproteobacteria*. In deeper anaerobic layers more diverse communities than in the upper layers were present. The deeper layers were dominated by *Spirochaetes*, sulfate-reducing bacteria (*Deltaproteobacteria*), Chloroflexi (Anaerolineae and Caldilineae), purple non-sulfur bacteria (*Alphaproteobacteria*), purple sulfur bacteria (*Chromatiales*), anaerobic *Bacteroidetes* (*Marinilabiacae*), *Nitrospirae* (OPB95), *Planctomycetes* and several candidate divisions. The archaeal community, including numerous uncultured taxonomic lineages, generally changed from *Euryarchaeota* (mainly *Halobacteria* and *Thermoplasmata*) to uncultured members of the *Thaumarchaeota* (mainly Marine Benthic Group B) with increasing depth.

## Introduction

Microbial mats are one of the best existing examples to study early life on Earth, as their fossil counterparts, stromatolites, could be dated back to approximately 3.5 Ga [1]–[3]. These very first ecosystems might have played a major role in forming our present atmosphere and thereby have paved the way for oxygen-dependent life [4], [5]. In general, microbial mats are vertically multilayered carpets of microbial assortments including specialized consortia of *Bacteria*, *Archaea*, and *Eukarya* that are embedded in self-produced extracellular polymeric substances (EPS). The metabolism of these communities results in physicochemical gradients leading to mat layering and changes in mineral saturation (for review see [6], [7]). Microbial mats are found in shallow aquatic environments all over the world such as thermal springs, hydrothermal vents, tidal flats, and hypersaline lakes [8]. Depending on the type, major functional groups in the microbial mats are photolithoautotrophs, aerobic/anaerobic heterotrophs, fermenters, sulfide oxidizers, and methanogens [7]. Some of these mats lead to the formation of microbialites, which are microbially induced mineral precipitations such as aragonite and gypsum. However, the processes of microbialite formation are still not fully understood [6].

Microbial mats were studied from Guerrero Negro (Baja California Sur, Mexico) [9]–[13], Highborne Cay (Bahamas) [14], [15], Shark Bay (Australia) [16], [17], and Solar Lake (Eilat, Israel) [18]. These studies revealed that microbial mats harbor a by far larger diversity of microbes than previously assumed. Furthermore, it has been shown that microbial mat communities possess a complex vertical distribution profile, which is mainly driven by light and oxygen penetration.

In this study, the microbial mat of the evaporitic, hypersaline Lake 21 located on Kiritimati atoll (Republic of Kiribati, Central Pacific) was investigated. This unique mineralizing mat is associated with the formation of decimeter-thick reticulate microbialites composed of aragonite and layers of gypsum crystals [19]. So far, initial studies regarding the hydrochemistry, microbial mat population and source of microbialite-formation have been conducted [19]–[22]. Recent studies analyzed the microbial community composition of the Kiritimati mats using microscopic observations, lipid biomarker analyses, bacteriohopanoids, or classical clonal 16S rRNA gene sequencing [19], [20], [22]. Initial 16S rRNA gene analysis revealed that bacteria affiliated to several phyla including *Cyanobacteria*, *Proteobacteria*, *Bacteroidetes*, *Firmicutes*, *Chloroflexi*, and *Planctomycetes*, and a small number of *Archaea* (*Euryarchaeota* and *Crenarchaeota*, most crenarchaeal members were reordered to *Thaumarchaeota* [23]) were present in the analyzed mat. Taking the small survey size of 359 16S rRNA gene sequences of previous studies and the high microbial diversity found in other microbial mats into account, the previous insights into the prokaryotic community composition were limited with respect to spatial distribution and coverage. Consequently, larger surveying efforts are required for the comprehensive description of the microbial communities in these ecosystems [9].

In this study, we investigated the bacterial and archaeal communities in nine different layers collected from a microbial mat of the hypersaline Lake 21. We analyzed approximately 197,000 16S rRNA gene sequences from *Bacteria* (122,000) and *Archaea* (74,000). Analysis revealed highly diverse microbial communities along the vertical stratification of the mat, which mirror the micro-environmental properties. The predominant bacterial and archaeal taxa of this ecosystem were identified. A large amount of the detected microbial taxa showed similarities to 16S rRNA gene sequences of so far uncultured organisms, suggesting the presence of novel species and metabolic traits in this habitat.

## Materials and Methods

### Site Description and Sample Collection

**Figure 1:**
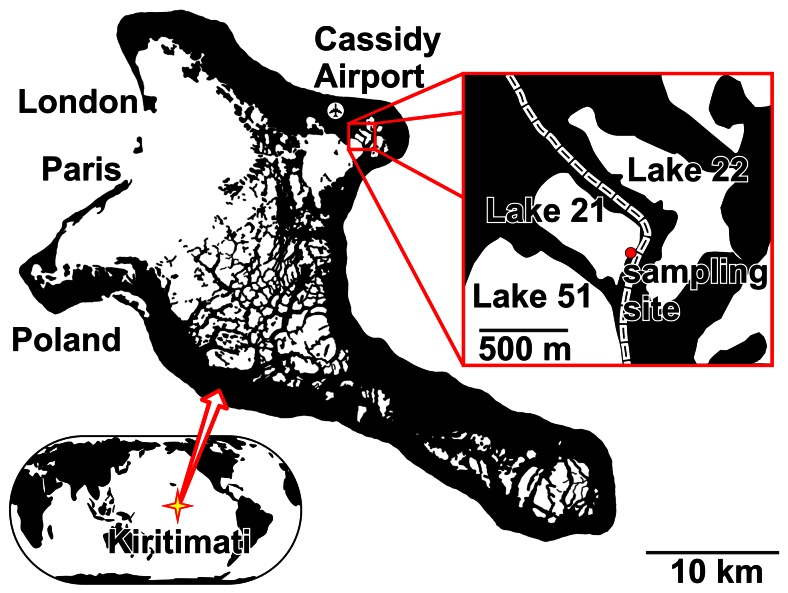
Map of Kiritimati Island and the sampling site at Lake 21.

Kiritimati (formerly Christmas Island) is the world's largest atoll and part of the Northern Line Islands of Republic of Kiribati with a land area of approximately 321 km^2^ [24]. Field work permits were kindly given by the responsible Ministry of Environment, Lands and Agricultural Development (MELAD) - Environment and Conservation Division, Republic of Kiribati (Bikenibeu, Tarawa, Republic of Kiribati). As microorganisms were studied, no experiments involving humans or vertebrates were conducted. Sampling of the microbial mat was carried out in March 2011 on the atoll of Kiritimati during a two-week expedition (Figure 1). The island harbors approximately 500 lakes with different salinities ranging from nearly freshwater to hypersaline conditions. The lakes are largely occupied by lithifying and non-lithifiying microbial mats [21], [25].

**Figure 2:**
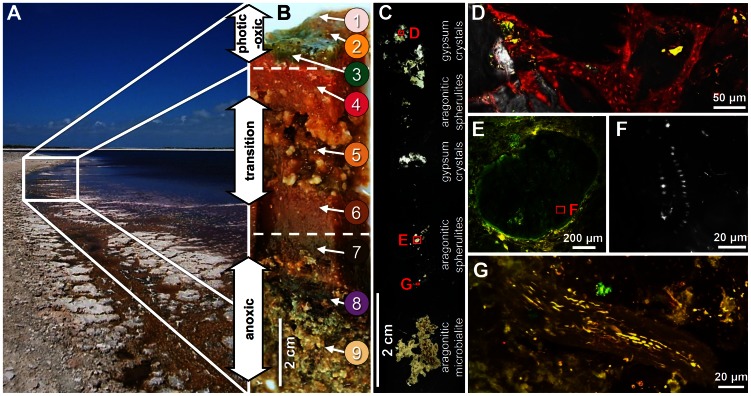
Localization of sampling site, structure, and characteristics of the microbial mat derived from Lake 21. Southwest view of the sampling site (A), cross-section of the microbial mat of Lake 21 (B), and vertical thin-section of the microbial mat and its mineral precipitates viewed under X-nicols (C). Detailed view of (C) showing *Cyanothece* with gypsum crystals from layer 3 (overlay of X-nicols and LSM image, ex 633 nm/em 640–702 nm) (D); spherulite with dead *Johannesbaptistia* from layer 7 (LSM micrograph ex 488, 543 and 633 nm/em 500–530, 565–615, 640–702 nm) (E); magnified subfossil *Johannesbaptistia* (LSM micrograph, ex 488 nm, em 500–550 nm) (F); ceased *Microcoleus* filament from layer 8 (LSM micrograph, ex 488, 543 and 633 nm/em 500–530, 565–615, 640–702 nm) (G). Description of microbial mat layers in (B): Layer 1 was a light pink, loose, and transparent film, scattered with black dots, which covered the mat only at the shoreline (0 to 2 m). Layer 2 was defined by bright orange coloration and could be observed in each sample. Layer 3 was green and layer 4 bright pink to red with small carbonate precipitations on top and was always located below the three surface layers. Layer 5 was a loose, slimy orange to brownish section interstratified with large gypsum crystals. Layer 6 was dark orange-brown with small carbonate precipitates at the bottom side. Layer 7 was dark brown and randomly seeded with small carbonate precipitates. Layer 8 was a dark purple layer, with reticulate microbialites. Layer 9 was creamy layer with sand like texture followed by a reticulate microbialite fabric [19].

The sampling site was located to the southeast shore line (1°96′N, 157°33′W) of the hypersaline Lake 21 which exhibited an average lake water salinity of 170‰. The lake was highly evaporated as samples were taken after a dry period of 11 months (Figure S1). Additionally, chimneys importing water with lesser salinity were observed in the middle of the lake. Lake water chemistry is depicted in Table S1. The structural profile of the microbial mat is well defined by a clear colored vertical zonation into nine layers (Figure 2). The mat surface was covered by 1 to 4 cm of lake water. The thickness of the mat was approximately 10 cm at the sampling site but reached up to 20 cm at the lake center. For each layer, four mat samples were collected at 3 pm in the described location. The mat was separated in the field into nine different layers, merged with RNALater (Qiagen, Hilden, Germany) to stabilize the nucleic acids. Subsequently, the samples were stored and transported frozen to Germany, where the samples were stored at −80°C until further use.

### Extraction of Microbial DNA

Equal amounts (1 g) of the four random samples for each of the nine mat layers were mixed and subsequently homogenized by grinding with liquid nitrogen to minimize potential sample heterogeneity. Environmental DNA was isolated of each pooled sample by employing the MoBio PowerBiofilm DNA isolation kit (MO BIO Laboratories, Carlsbad, USA) following the guidelines of the manufacturer with slight modifications. For each DNA-isolation, 100 mg sample was used. The volume of buffer 3 was increased two-fold as described in the manual for strong contaminated samples. Final purification and concentration of DNA was achieved by employing SureClean (Bioline GmbH, Luckenwalde, Germany) as recommended by the manufacturer. The DNA yield was determined by using a NanoDrop ND-1000 spectrophotometer (Peqlab Biotechnologie GmbH, Erlangen, Germany).

### Amplification of 16S rRNA Genes and Pyrosequencing

The hypervariable regions V3 to V5 of the 16S rRNA gene were amplified by PCR. For amplification of 16S rRNA genes the PCR reaction mixture (50 µl) contained 10 µl of 5-fold reaction buffer (Fusion GC buffer, Finnzymes, Vantaa, Finland), 200 µM of each of the four deoxynucleoside triphosphates, 0.2 µM of each primer, 5% DMSO (Finnzymes), 1 U of Phusion hot start high-fidelity DNA Polymerase (Finnzymes) and 50 ng of isolated DNA as template. Primer sequences for amplification of the V3 to V5 region included the Roche 454 pyrosequencing adaptors (underlined), a key (TCAG), and a variable multiplex identifier (MID) consisting of ten bases. For *Bacteria* the forward primer used was V3for_B 5′-CGTATCGCCTCCCTCGCGCCATCAG-MID-TACGGRAGGCAGCAG-3′ [26] and the reverse primer V5rev_B 5′-CTATGCGCCTTGCCAGCCCGCTCAG-MID-CCGTCAATTCMTTTGAGT-3′ [27]. For amplification of archaeal 16S rRNA genes the forward primer V3for_A 5′-CGTATCGCCTCCCTCGCGCCATCAG-MID-CCCTAYGGGGYGCASCAG-3′ [28] and the reverse primer V5rev_A 5′-CTATGCGCCTTGCCAGCCCGCTCAG-MID-GTGCTCCCCCGCCAATTCCT-3′ [29] were used. Taxonomic coverage of selected primer pairs was determined for *Bacteria* and *Archaea* with PrimerProspector [30] (Figure S2). The following thermal cycling scheme was used for amplification of partial bacterial 16S rRNA genes: initial denaturation at 98°C for 5 min, 25 cycles of denaturation at 98°C for 45 s, annealing for 45 s at 60°C, and extension at 72°C for 30 s, followed by a final extension period at 72°C for 5 min. For amplification of the archaeal 16S rRNA genes, the annealing temperature was adjusted to 57°C. Negative controls contained the entire reaction mixture without the template DNA. The resulting PCR products were checked by agarose gel electrophoresis for appropriate size and purified by gel extraction using the peqGold gel extraction kit as recommended by the manufacturer (Peqlab Biotechnologie GmbH). Quantification of the PCR products was performed using the Quant-iT dsDNA BR assay kit and a Qubit fluorometer (Invitrogen GmbH, Karlsruhe, Germany) as recommended by the manufacturer. All PCR reactions were performed in triplicate and pooled in equal amounts.

The 16S rRNA gene sequences were deposited in the National Center for Biotechnology Information (NCBI) Sequence Read Archive (SRA) under project accession number SRA058120.

### Processing and Analysis of 16S rRNA Gene Datasets

The Göttingen Genomics Laboratory determined the sequences of the partial 16S rRNA gene amplicons by using a 454 GS-FLX pyrosequencer (Roche, Mannheim, Germany) and Titanium chemistry following the instructions of the manufacturer for amplicon sequencing. Generated 16S rRNA gene datasets were processed and analyzed employing the QIIME 1.4 software package [31]. Initially, we removed sequences shorter than 250 bp, containing unresolved nucleotides, exhibiting an average quality score lower than 25, harbor mismatches longer than 2 bp in the forward primer, or possessing homopolymers longer than 8 bp with the QIIME script *split_libraries.py*. This also removes forward and reverse primer sequences. Subsequently, pyrosequencing noise was removed by employing the Denoiser 0.91 [32] included in QIIME [31]. We additionally removed unclipped reverse primer sequences (not detected by *split_library.py*) by employing cutadapt [33] with default settings. Chimeric sequences were removed using UCHIME [34] included in USEARCH (6.0.152). As the studied environment might harbor *Bacteria* and *Archaea*, which are not well represented by reference databases, we applied UCHIME in de novo mode. Subsequently, we used UCHIME in reference mode against the Greengenes Gold dataset (gold_strains_gg16S_aligned_19-Mar-2011.fasta) as the reference database [34], [35]. Operational taxonomic unit (OTUs) determination was performed with the UCLUST algorithm [36] at a genetic divergence level of 3%, which represents species-level according to Schloss and Handelsman [37]). For this purpose, most abundant sequence within an OTU was picked by employing the QIIME scripts *pick_otus.py* and *pick_rep_set.py* [31]. OTUs clustered at 3% genetic divergence were the basis of all presented results. Taxonomic classification of the picked reference sequences (OTUs) was performed by similarity searches using BLAST (blastall version 2.2.18) [38] against the SILVA SSU database release 111 (SSURef_111_NR_tax_silva_trunc.fasta) [39]. The SILVA taxonomy of the first hit based on E-value was then adopted to infer taxonomy of the representative sequences according to the QIIME script *assign_taxonomy.py*. An OTU table was created using *make_otu_table.py*. Singletons, chloroplast and extrinsic domain OTUs were removed from the table by employing *filter_otu_table.py*. Details on the sequence data processing are given in Table S2. Finally, OTUs that exhibited low coverage (<95%) to their BLAST hit were designated as “Unclassified”.

### Diversity Indices and Rarefaction Analysis

The alpha-diversity indices such as Chao1 indices, Shannon indices, and Michaelis-Menten fit were calculated employing the QIIME script alpha_diversity.py *(10 replicates per sample) (* *Table 1* *). Shannon indices were multiplied by ln 2 to allow comparison with non-QIIME-based studies. Rarefaction curves were calculated from the modified OUT table with alpha_rarefaction.py* (step size 50 for *Bacteria* and 25 for *Archaea*; 10 replicates per sample). *The analyses were performed* at the same level of surveying effort (6,350 bacterial and 2,350 archaeal randomly selected sequences per sample).

**Table 1:** Bacterial and archaeal 16S rRNA gene diversity analyses of microbialite-forming Lake 21 mat at 3% genetic divergence level.

Sample	No. high quality reads	No. OTUs	Chao1	Shannon	Michaelis Menten fit	Coverage (%)
***Bacteria***						
Layer 1	9,445	95	135.8	2.01	111.6	85.4
Layer 2	6,646	67	79.7	2.43	69.4	96.9
Layer 3	6,315	98	130.6	2.77	101.9	96.1
Layer 4	6,279	142	166.2	3.32	154.8	91.7
Layer 5	7,505	163	184.3	3.68	172.6	94.4
Layer 6	12,580	145	174.9	3.36	155.9	93.2
Layer 7	9,741	227	275.1	4.10	248.9	91.2
Layer 8	10,417	273	317.2	4.37	299.6	91.0
Layer 9	11,117	199	242.0	3.34	226.8	87.6
***Archaea***						
Layer 1	3,107	28	29.1	1.53	30.1	93.1
Layer 2	3,805	22	33.6	1.16	25.0	89.0
Layer 3	3,418	27	32.3	1.33	29.3	91.4
Layer 4	3,524	27	35.6	1.47	31.6	84.9
Layer 5	10,663	26	33.6	1.91	26.1	98.0
Layer 6	7,235	36	52.4	1.65	42.3	86.0
Layer 7	3,080	33	41.9	1.44	37.0	88.6
Layer 8	2,544	41	48.1	2.11	43.0	96.1
Layer 9	2,353	27	33.0	1.37	33.2	81.2

### Community Comparison of Microbial Mat Layers

To compare the bacterial and archaeal communities across all mat layers a principal component analysis (PCA) was performed using Canoco 4.56 (Microcomputer Power, Ithaca, NY, USA). The relative abundances of bacterial phyla, candidate divisions, proteobacterial subclasses, archaeal families and candidate groups, unclassified groups, and the artificial group “Other” (summary of all phyla, subdivisions and candidate divisions, accounting for less than 0.5% relative abundance in any given sample) were used. The scaling was focused on inter-taxa correlations and taxa scores were divided by standard deviation. The graph was centered by taxa.

For the bacterial community comparison of Guerrero Negro and Kiritimati microbial mat layers, the Guerrero Negro 16S rRNA clone library gene sequences of the ten analyzed layers were obtained from GenBank (Bioproject PRJNA29795). Sequences were trimmed to the corresponding 16S rRNA gene region (V3–V5) of the Kiritimati sequences by employing the FASTX-Toolkit (http://hannonlab.cshl.edu/fastx_toolkit/). Sequences were further processed as described for the Kiritimati sequences. Weighted principle coordinate analysis (PCoA) was performed at the same level of surveying effort employing the QIIME scripts *beta_diversity_through_plots.py* and *make_3d_plots.py* for plotting OTUs to the weighted PCoA.

### Lake and Pore Water Hydrochemistry

Temperature, electrical conductivity, pH, and redox potential of lake and pore waters were recorded *in situ* using portable instruments from WTW GmbH, Germany. The utilized Schott pH-electrode was calibrated against NBS buffers 7.010 and 10.010 (HANNA instruments). Total alkalinity was immediately determined after sampling by acid-base titration using a hand-held titrator and 1.6 N H_2_SO_4_ cartridges as titrant (Hach Corporation). Dissolved oxygen was analyzed titrimetrically following the Winkler method. Samples for determination of main anions and cations were collected in pre-cleaned PE-bottles. Samples for cation analysis were filtered in the field through 0.8 µm membrane filters (Millipore) and fixed by acidification. Main cations (Ca^2+^, Mg^2+^, Na^+^, and K^+^) and anions (Cl^−^ and SO_4_^2−^) were analyzed by ion chromatography with conductivity detection (Dionex Corporation and Metrohm). ICP-OES (Perkin Elmer) was used to determine Sr^2+^ and Fe^2+^. Dissolved phosphate, silica, and ammonia concentrations were measured by spectrophotometric methods (Unicam). Measured values were processed with the computer program PHREEQC [40] in order to calculate ion activities and *P*CO_2_ of the water samples as well as saturation state with respect to calcite, aragonite and gypsum.

## Results and Discussion

### Characteristics of the Lake 21 Microbial Mat

**Figure 3:**
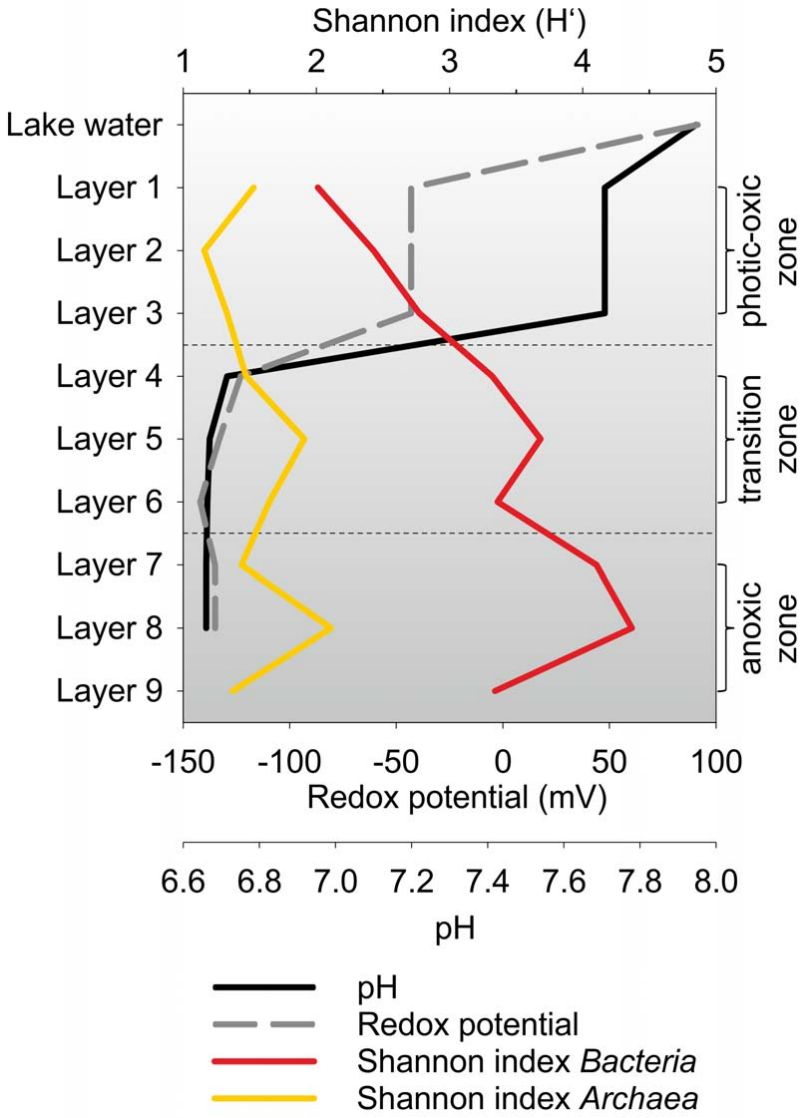
Hydrochemical data of lake and pore water and Shannon indices of sampled microbial mat layers. The redox potential (Eh in mV) and pH (at 32°C) were measured in the field (Table S3). Shannon indices were calculated at 3% genetic divergence and same level of surveying effort (Table 1).

The microbialite-forming mat samples were taken from the shoreline of the southeastern part of Lake 21 (Figure 1). The lake water has been analyzed with respect to physical and physicochemical parameters, cations, anions and nutrients (Table S1). The hydrochemical data of lake and pore waters are depicted in Figure 3. The water chemistry of the lake was characterized by a pH of 7.95, a temperature of 32°C and an average salinity of 170‰. Cation and anion concentrations of the lake water were 2.6 M Na^+^, 3.1 M Cl^−^, 298.3 mM Mg^2+^, and 143.1 mM SO_4_^2−^. These data correspond to the data gained by Arp *et al.* in 2002, but ion concentrations were approximately 1.6-fold higher in 2011. This might be a result of strong evaporation of the lake water caused by a dry period of almost one year prior to sampling in 2011 (Figure S1). Lake 21 pore waters generally show high Ca^2+^ and SO_4_^2−^ concentrations, which are probably caused by surface water or groundwater [19]. Major ion supply to the lake water is currently due to the influx of less saline groundwater (Table S1) via chimney-like microbial mat structures. With respect to salinity, the hypersaline Lake 21 was more similar to the Solar Lake (salinity 200‰) than to Guerrero Negro (salinity 90‰) [9], [11], [18].

We observed nine color-separated mat layers with fractional carbonate precipitations (Figure 2B). A reticulate microbialite was observed below the microbial mat, as described earlier in 2002 [19]. In contrast to our study, Arp *et al.* found 2002 a separation in only four layers, which exhibited orange, green, purple, or grey/brown color [19]. The below-described phylogenetic analysis indicated that these four layers were overgrown by a new mat community and represented layers 6 to 9 in the present microbial mat.

The nine microbial mat layers were separated and analyzed. The chemo-physical properties of the nine mat layers and the arrangement of the detected phylogenetic groups indicated roughly a separation into three major mat zones. These zones were designated photic-oxic zone (layers 1–3), transition zone (dysphotic, low oxygen, layers 4–6), and anoxic zone (layers 7–9) with respect to their main characteristics (Figure 3). The pH measurements during daytime showed slightly alkaline pH value in the lake water (7.95) and the photic-oxic zone (7.7) and slightly acidic pH values in the transition (6.68) and anoxic (6.66) zones. Similar observations were made for the Guerrero Negro mat, but the pH range was broader (pH range of approximately 8.2 to 6.2 from top to bottom) [11]. The redox potential in Lake 21 mat changed from positive in the lake water (+91.4 mV) to negative in the photic-oxic zone (−43 mV). Within the mat layers, a redox potential gradient ranging from −43 mV (photic-oxic zone) to −135 mV (anoxic zone) was recorded. This gradient is typical for microbial mat systems, as the conditions change from oxic to anoxic with increasing depth.

### Diversity and General Characteristics of the Bacterial Community

Two studies previously investigated the microbial communities of the Lake 21 mat. One study focused on the analysis of bacteriohopanepolyols (BHP) within the mat, which were classified as cyanobacterial and proteobacterial BHPs [22]. The second study identified microbes by Sanger-based sequencing of clonal 16S rRNA genes [19]. However, due to low phylogenetic resolution of the BHP analysis and small survey size (395 sequences) of the other study, a detailed and comprehensive assessment of the microbial mat community is still lacking. In this study, large-scale next-generation 16S rRNA gene sequencing was used to increase the survey size with respect to the vertical stratification. As indicated by metagenomic and molecular studies of other microbial mats, bacteria are the predominant life forms in these habitats followed by archaea and eukaryotes [10], [41]–[44]. The bacterial 16S rRNA gene analysis resulted in recovery of 80,045 high quality sequences with an average read length of 429 bp. The number of sequences per layer ranged from 6,279 (layer 4) to 12,580 (layer 6). We were able to assign all 16S rRNA gene sequences to the domain *Bacteria* and to classify 78,742 of these sequences below the domain level. The classified sequences were affiliated to 26 bacterial phyla and candidate divisions. Dominant bacterial groups that showed more than 0.5% abundance in any layer belonged to 11 phyla and 4 candidate divisions. In addition, the bacterial community composition showed a well-defined stratification along the depth gradient of the mat.

**Figure 4:**
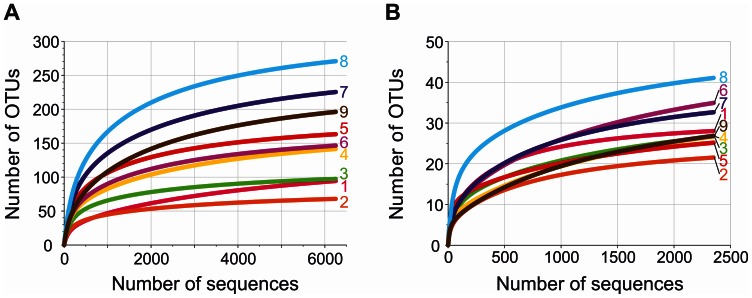
Rarefaction analysis of the bacterial (A) and archaeal (B) communities in the mat layers. Curves were calculated at the same level of surveying effort at 3% genetic divergence employing QIIME [31].

**Figure 5:**
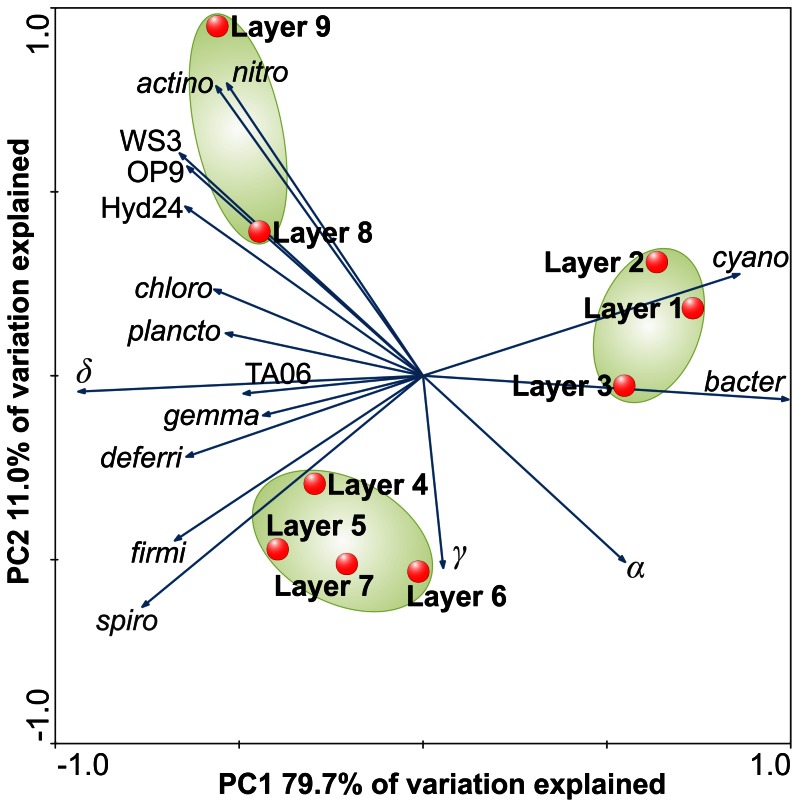
PCA of bacterial communities in the mat layers. PCA was based on relative abundances of bacterial phyla, proteobacterial classes, and candidate divisions. Blue arrows point in direction of increased taxon abundance. Clustering of sampling sites due to similarities in bacterial community composition is highlighted by green ellipses. Abbreviations: *α*, *Alphaproteobacteria*; *β*, *Betaproteobacteria*; *γ*, *Gammaproteobacteria*; *δ*, *Deltaproteobacteria*; *actino*, *Actinobacteria*; *nitro*, *Nitrospirae*; OD1, Candidate division (CD) WS3, CD WS3; Hyd24, CD Hyd24; TA06, CD TA06; *chloro*, *Chloroflexi*; *plancto*, *Planctomycetes*; *gemma*, *Gemmatimonadetes*; *deferri*, *Deferribacteres*; *firmi*, *Firmicutes*; *spiro*, *Spirochaetes*; *bacter*, *Bacteroidetes*; *cyano*, *Cyanobacteria*.

To determine the bacterial diversity and richness rarefaction analyses were performed. Alpha diversity analysis was performed at the same level of surveying effort. The *Bacteria* encompassed 422 OTUs at 3% genetic divergence level. The OTU number in the layers ranged from 67 (layer 2) to 273 (layer 8). In general, the bacterial diversity increased with depth (Figure 3). This has also been reported from other microbial mat studies such as Guerrero Negro and Solar Lake [9], [18]. In the top layers diversity estimates could also be influenced by sedimentation of planktonic bacteria that live in the water column. For example, *Salisaeta longa*, which is the closest related cultured representative of the most abundant OTU in layer 1 (56% of the total bacterial community), was isolated from hypersaline water bodies [45]. Rarefaction curves generally showed saturation for the bacterial communities of all nine samples at 3% genetic divergence (Figure 4A). The maximal number of expectable OTUs was estimated for each sample by using the Michaelis-Menten fit alpha diversity estimation. The average coverage was 91.9%. Thus, the majority of bacterial phylotypes was covered by the surveying effort. Shannon indices ranged from 2.01 to 4.37 with maxima in layers 5 and 8 (Figure 3). Direct comparison of the bacterial mat layer communities by PCA revealed three clusters (Figure 5). The top cluster the middle cluster, and the bottom cluster consisted of layers 1 to 3, layers 4 to 7, and layers 8 to 9, respectively.

### Bacterial Community Composition and Distribution

**Figure 6:**
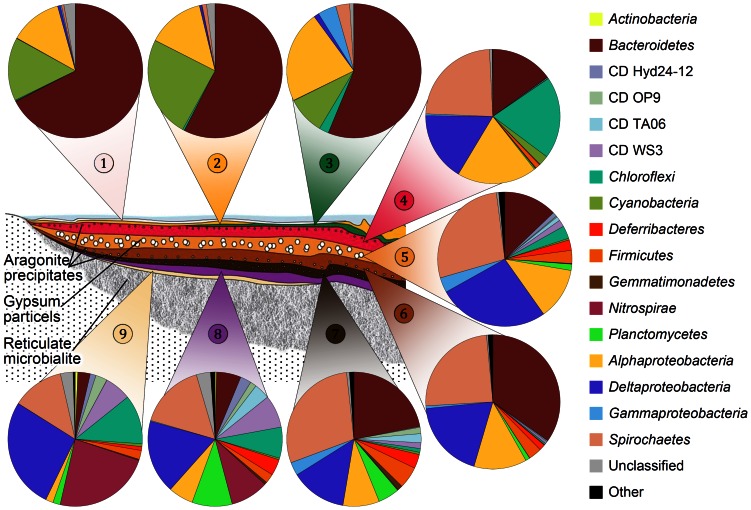
Model of the stratified microbial mat of Lake 21 and relative abundances of bacterial phyla. Illustrated are the bacterial phyla, candidate divisions, and proteobacterial classes found within the different layers. Taxonomic classification was performed according to SILVA SSU rRNA database 111 [39]. Bacterial phyla with a relative abundance lower 0.5% were summarized as the artificial group “Other”. The artificial group “Other” includes *Acidobacteria*, CD BHI80-139, CD BRC1, CD JL-ETNP-Z39, CD NPL-UPA2, CD WS6, *Chlorobi*, *Deinococcus*-*Thermus*, *Fibrobacteres*, *Lentisphaerae*, and *Synergistetes* (for details see Figure S3).

The bacterial community was dominated by the phyla *Bacteroidetes* (30.5%), *Proteobacteria* (27.3%), *Spirochaetes* (15.3%), *Cyanobacteria* (5.9%), *Chloroflexi* (5.2%), *Nitrospirae* (3.7%), *Planctomycetes* (2.2%), *Firmicutes* (1.9%), CD WS3 (2%), unclassified taxa (1.6%), and *Deferribacteres* (1.4%) (Figure 6). The following phyla and candidate divisions represented each less than 1% of the total bacterial communities *Gemmatimonadetes*, *Fibrobacteries*, *Acidobacteria*, *Actinobacteria*, *Lentisphaerae*, *Deinococcus*-*Thermus*, *Chlorobi*, *Synergistetes*, and several candidate divisions (OP9, TA06, Hyd24-12, BRC1, NPL-UPA2, BHI80-139, WS6, and JL-ETNP-Z39) (Figure S3).

The photic-oxic zone was represented by the surface layers 1, 2 and 3. In general, the bacterial communities of these layers were highly similar. The zone harbored *Cyanobacteria* as primary producers of organic matter. One predominant phylotype was detected that showed high 16S rRNA gene similarity (98%) to an unicellular, extreme halotolerant species of the *Halothece* cluster (*Euhalothece* sp. strain MPI 95AH13, AJ000710), which was isolated from a benthic gypsum crust in a solar evaporation pond (Eilat, Israel) [46]. The presence of this phylotype has also been observed in lithifying and non-lithifying hypersaline microbial mats, e.g. in the Guerrero Negro hypersaline mat (JN454085) [9], [14], [16], [47], [48]. Other detected cyanobacterial lineages in the photic-oxic zone were *Spirulina* but only in minor abundances (<1%, mainly layer 4). *Cyanobacteria* were detected in significant abundances, but exhibited a lower diversity than in other saline microbial mats such as Guerrero Negro or Highborne Cay [9], [14]. Studies of extreme halophile *Cyanobacteria* showed that high salinity precludes growth of certain cyanobacterial species such as *Synechococcus* sp. (AJ000716), as most unicellular *Cyanobacteria* prefer growth at <130‰ salinity [46]. Correspondingly, we observed in a microbial mat from the less saline Kiritimati Lake 2 (salinity approximately 120‰) that the cyanobacterial diversity was higher than in the Lake 21 microbial mat (data not shown). The *Euhalothece*-related *Cyanobacteria* were accompanied with known phototrophic or putative phototrophic members of the *Alphaproteobacteria* and *Bacteroidetes*. The *Alphaproteobacteria* of the surface layers were mainly represented by purple non-sulfur bacteria, which were classified as uncultured members of *Rhodobacterales* and *Rhodospirillales*. These were also detected in the surface layers of the Guerrero Negro and Solar Lake mats [9], [18]. The 16S rRNA gene sequences of the *Rhodobacterales* showed similarities to uncultured *Rhodovulum*, *Sediminimonas*, and several uncultured *Rhodobacteraceae*. The low similarities (<95%) of these 16S rRNA genes to cultured species indicated that several novel *Rhodobacterales* species may thrive in the surface part of the mat. Most *Rhodospirillaceae* prefer anoxic conditions, are photoheterotrophic in light and grow chemotrophically in dark. This suggests that anoxic niches exist in the surface mat matrix (in accordance with the diel cycle) or that the here occurring species differ in their characteristics from known species [49]. *Bacteroidetes*, which predominated the surface layers, consisted of uncultured members of the *Rhodothermaceae* (mainly uncultured *Salisaeta* and *Salinibacter*) and *Saprospiraceae*. *Saprospiraceae* and *Salinibacter* increased in abundance from layer 1 to layer 3, whereas *Salisaeta* decreased. The localization in the upper layers suggests phototrophy for these bacterial groups. *Salinibacter* species have been detected in several hypersaline environments around the world, such as lakes, and coastal or solar salterns [50]. Correspondingly, the genome of the extremely halophilic, photoheterotrophic *Salinibacter ruber* isolated from a microbial mat contains four rhodopsins [51]. Interestingly, three of these were halorhodopsins that typically occur in the domain of *Archaea*. Rhodopsin-containing *Archaea* belonging to the *Halobacteria* have been detected in the surface layers (see below), indicating the possibility of horizontal gene transfer (HGT) events between archaea and bacteria. Recently, considerable numbers of (halo-)viruses have been detected in hypersaline habitats, which could be an additional source for gene transfer events [52].

The transition zone spans approximately layers 4 to 6. In this zone, the most drastic change of the microbial community composition was observed. Simultaneously, the bacterial diversity increased (approximately 1.4-fold) compared to the photic-oxic zone. This alteration was observed on phylum and subgroup level. The community in this zone was dominated by *Proteobacteria*, *Spirochaetes*, *Bacteroidetes*, *Chloroflexi*, *Firmicutes* and *Deferribacteres*. The composition of the *Alphaproteobacteria* and *Bacteroidetes* subgroups changed compared to the photic-oxic zone. The *Alphaproteobacteria* were mainly represented by the *Rhizobiales*. The predominant alphaproteobacterial OTU was an uncultured *Dichotomicrobium*, which was abundant in layers 3 to 8. The 16S rRNA gene sequence of this OTU exhibited high similarity (99%) to *Dichotomicrobium thermohalophilum* (FR733679), which is an aerobic, moderate thermophilic and halophilic heterotroph isolated from the hypersaline Solar Lake (Eilat, Israel). This species builds net-like hyphae and thereby may also be a structural component of the microbial mat fabric [53]. In layer 4, low numbers of 16S rRNA gene sequences affiliated to *Roseospira* (1.5%) and *Rhodovibrio* (1.1%) were observed in addition to uncultered *Rhodospirillales* (2.5%). *Roseospira* and *Rhodovibrio* disappeared in the lower layers. *Bacteroidetes* appeared in layer 4 in low abundance and were represented by *Rhodothermaceae* and *Marinilabiaceae*. The latter group was encountered in layers 4 to 7, with highest abundance in layer 6 (27.6% of the total bacterial community), indicating a preferred anaerobic lifestyle. The dominant OTU of the *Marinilabiaceae* possessed highest sequence similarity (approximately 96%) to a Guerrero Negro mat clone (JN445925). In addition, *Chloroflexi*, *Deltaproteobacteria* and *Spirochaetes* occurred in the transition zone. *Chloroflexi* were a major component of layer 4, and consisted primarily of uncultured members of *Anaerolineaceae*, which exhibited similarities to 16S rRNA gene sequences of *Anaerolineaceae* derived from the Guerrero Negro mat. Members of this family are strictly anaerobic, filamentous, multicellular, non-pigmented and non-phototrophic [54]. The *Deltaproteobacteria* accumulated in the transition zone and formed a main part of the bacterial community through the deeper mat layers. The majority of the detected representatives of the *Deltaproteobacteria* such as *Desulfovibrionales*, *Desulfobacterales*, and *Desulfarculales* are sulfate-reducing bacteria (SRB). SRB play a major role in carbon cycling and evolved before oxygenic photosynthesis developed [55]. As the reduction of sulfate is an anaerobic process, the distribution of SRB in the transition and anoxic zone corresponds well with the mat properties. However, traces of *Deltaproteobacteria* (0.6 to 1.2%) were observed in the photic-oxic zone. Recently, it has been shown that SRB in microbial mats possess oxygen tolerance [20], [55]. Furthermore, it has been demonstrated that some *Desulfovibrio* not only tolerate but also use low oxygen tensions for growth [56], [57]. The *Desulfobacterales* were primarily composed of uncultured members of *Desulfobacteraceae* and a small number of *Desulfosalsimonas*, which both have been previously isolated from microbial mats [9], [58]. The *Desulfovibrionales* were affiliated to uncultured *Desulfovibrio* and *Desulfovermiculus* (>98% 16S rRNA gene sequence similarity to *Desulfohalobium utahense*). *Desulfohalobium utahense* was isolated from the Great Salt Lake (Utah, USA) and is characterized as an anaerobic chemoorganotrophic SRB [59]. *Spirochaetes* also formed a major part of the center and bottom mat communities and were highly diverse (45 OTUs). All 16S rRNA gene sequences were affiliated to uncultured *Spirochaeta,* which were also detected in the Guerrero Negro mats [9]. Their cork screw-like type of movement might be advantageous in EPS-rich mat habitats, as flagella mediated movement might be less efficient. An interesting result was the unique occurrence of purple sulfur bacteria (PSB) affiliated to the *Chromatiales* in layer 5, which was characterized by abundant spherical gypsum crystals (Figure 2C). Members of the PSB, *Chromatiaceae* (2.3%) and *Ectothiorhodospiraceae* (1%), were detected in low abundances within layer 5. PSB of these families are phototrophic and produce internal (*Chromatiaceae*) and external (*Ectothiorhodospiraceae*) elemental sulfur globules, respectively [60]. The preferential occurrence in one layer in addition with the metabolic features of the PSB might point to an involvement in gypsum precipitation. The relative low abundance of this group could be caused by low recovery of DNA from these organisms due to adherence of cells to gypsum crystal fissures.

Finally, the anoxic light-free zone (layers 7 to 9) harbored mainly *Spirochaetes*, *Deltaproteobacteria*, *Gammaproteobacteria*, *Planctomycetes*, *Nitrospirae*, *Firmicutes*, *Deferribacteres*, and different candidate divisions (WS3, TA06, OP9, Hyd24-12). These phyla showed specific patterns within their composition along the depth gradient. *Deltaproteobacteria* composition changed from *Desulfobacterales* and *Desulfovibrionales* in the transition zone to *Desulfobacterales* (more diverse, including *Nitrospinacaea* and candidate group SEEP-SRB1) and *Desulfarculales* in the anoxic zone. The *Desulfarculales* were primarily represented by an OTU, which was detected in an earlier molecular study of the same lake (HM480235) [19]. The genome of the sulfate-reducing *Desulfarculus barsii* indicates that members of this family are strictly anaerobic, non-fermentative and chemoorganotrophic, and oxidize organic substrates completely to CO_2_ [61]. The *Chloroflexi* appeared in low abundances (0.9% to 3.1%) in layers 5 to 7, but increased in abundance in layers 8 and 9 (7.4% and 11.9%, respectively). In detail, low amounts of *Anaerolineales* were present, but mainly *Caldilineales* and several candidate subgroups were detected. These subgroups included MSBL5 (Mediterranean Sea Brine Lake group 5) and GIF9 (Groundwater In Flow clone 9), which were originally found in deep anoxic hypersaline Bannock basin and a contaminated groundwater-treated reactor, respectively [62], [63]. In addition, uncultured members of the *Nitrospirae*, in particular the candidate order OPB95 (formerly candidate division OP8), increased in abundance in layers 8 and 9. Members of this group were first encountered in Obsidian Pool (Yellowstone, USA) and could be involved in hydrogen oxidation and sulfate reduction [64]. Interestingly, the most abundant *Nitrospirae* OTU showed high 16S rRNA gene sequence similarity (99%) to a 2002 amplified 16S rRNA gene of the same mat (HM480241). Another group inhabiting the last three layers with highest abundance in layer 8 was *Planctomycetes*, composed of candidate Pla4 lineage and candidate group vadinHA49. The dominant OTU of the Pla4 lineage showed low similarity (<92%) to a Guerrero Negro mat clone (JN510021), which was also found in deeper layers (10–22 mm). The clone PLA4 (AF525959), which is one of the reference sequences for the Pla4 lineage, was found in a biological contactor fed with NH_4_^+^ wastewater [65], suggesting that the related microbes in Lake 21 could also be involved in (anaerobic) nitrogen cycling. *Firmicutes* were encountered in low abundances in the transition to anoxic zones of the mat and showed highest abundance in layers 7 (5.3%). Nevertheless, with 38 OTUs the *Firmicutes* were a diverse group that was primarily comprised of *Clostridia* belonging to the *Clostridiales* (mainly *Ruminococcaceae*) and *Halanaerobiales* (*Haloanaerobiaceae*). *Halanaerobiales* are anaerobic, halophilic and fermenting microorganisms [66]. Their lifestyle corresponds well with their location in the deeper anoxic parts of the mat. Members of this order have also been detected in deeper zones of hypersaline endoevaporitic microbial mats from the Solar Lake (Eilat, Israel) [18]. *Deferribacteres* showed a similar distribution as the *Firmicutes*. The middle layers were mainly inhabited by members of the candidate group LCP-89, which was also detected in saltmarsh sediment contaminated with mercury and PCB. In the lower layers, also uncultured *Caldithrix* relatives were present. These are known as anaerobic and chemoorganotrophic organisms, which are able to perform nitrate reduction and fermentation of di- and polysaccharides [67], [68]. However, the 16S rRNA gene sequence similarity to cultured representatives like *Caldithrix abyssi* was rather low (85%). Several members of the candidate phyla WS3, TA06, OP9, Hyd24-12 were encountered in the deeper layers. Especially WS3 and TA06 were abundant in the last three layers. The increase of candidate phyla in the deeper anoxic layers probably originates from tight metabolic dependencies on spatial surrounding species.

### Archaeal Diversity and Stratification in Microbial Mat Communities

**Figure 7:**
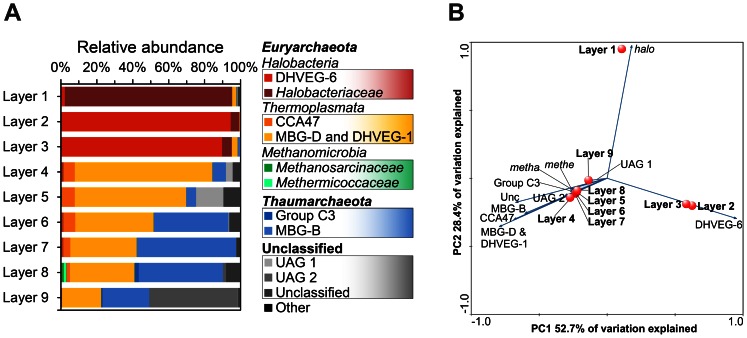
Relative abundances and comparison of the archaeal communities of the Lake 21 microbial mat. Relative abundances of archaeal lineages identified within the different microbial mat layers (A). Taxonomic classification was performed according to SILVA SSU rRNA database 111 [39]. Taxonomic lineages with a relative abundance lower 0.5% were summarized as the artificial group “Other”. Abbreviations: DHVEG-6, Deep Sea Hydrothermal Vent Group 6; CCA47, deep anoxic layer of an intertidal pool at Cape Cod; MBG-D and DHVEG-1, Marine Benthic Group D and Deep Sea Hydrothermal Vent Group 1; Group C3, Crenarcheotic Group C3 from marine lakes and sediments; MBG-B, Marine Benthic Group B; Other: Miscellaneous Euryarchaeotic Group; 20c-4, marine sediment (Greece, Aegean Sea); TMEG, Terrestrial Miscellaneous Group; MCG, Miscellaneous Crenarchaeotic Group (for details see Figure S4). Comparison of archaeal communities of microbial mat layers by PCA (B). PCA was based on relative abundances of archaeal lineages. Blue arrows point in direction of increased taxon abundance. Abbreviations: *halo*, *Halobacteriaceae*; DHVEG-6, see (A), MBG-D & DHVEG-1, Marine Benthic Group D and Deep Sea Hydrothermal Vent Group 1; CCA47, see (A); MBG-B, see (A); Group C3, see (A); Unc, unclassified archaeal groups; UAG 1, Unclassified archaeal group 1; UAG 2; unclassified archaeal group 2; *metha*, *Methanosarcinaceae*; *methe*, *Methermicoccaceae*.

Recent studies indicate that *Archaea* account only for a small part of microbial mat communities. Nevertheless, they may have important roles in biogeochemical cycles. The archaeal community analysis was carried out for the nine layers of the Lake 21 mat and resulted in 39,729 high-quality 16S rRNA gene sequences with an average read length of 513 bp. The number of sequences per layer ranged from 2,353 (layer 9) to 10,663 (layer 5). All 16S rRNA gene sequences could be assigned to the domain *Archaea* (Table 1). The trend of increasing diversity downwards the microbial mat persisted, but not as pronounced as for *Bacteria*. Interestingly, Shannon indices for archaeal diversity showed a similar depth profile to that of *Bacteria*, but overall diversity is approximately 2.1-fold lower (Figure 3). In addition, the highest degree of archaeal diversity with respect to Shannon index and OTU count was also detected in layers 5 and 8. A total of 86 different archaeal OTUs at 3% genetic divergence could be observed. The amount of OTUs in the different layers ranged from 22 (layer 2) to 41 (layer 8). Rarefaction curves generally showed saturation for all samples (Figure 4B). A similar trend was also observed for *Bacteria* (Figure 4A). The coverage of the archaeal communities was 89.8% on average. Thus, the majority of *Archaea* was detected by the surveying effort. Comparison by PCA of the archaeal community composition of all layers showed separation of the surface layers (layers 1 to 3) and the bottom layers (layers 4 to 9), which resembles the change from aerobic to anaerobic archaeal community members (Figure 7B).

The archaeal phyla *Euryarchaeota* and *Thaumarchaeota* were detected and represented 67.6 and 20.8% of all archaeal 16S rRNA gene sequences, respectively (Figure 7A). Additionally, due to low alignment coverage (<95%) several OTUs could not be classified below the domain level. Two of these were high abundant and designated as unclassified archaeal groups (UAG) 1 and 2. These groups occured in layer 5 (UAG1) and layer 9 (UAG2). UAG1 and UAG2 showed highest identities (85 and 82%, respectively) to 16S rRNA gene sequences from uncultured *Euryarchaeota* (DHVEG-6) (AACY020254990) and uncultured *Thaumarchaeota* (MBG-B) (AB644695), respectively. In general, *Euryarchaeota* dominated the layers 1 to 6, whereas *Thaumarchaeota* were generally present from layers 4 to 9 and exhibited highest abundances in layers 7 and 8 (Figure 7B). The archaeal community of the photic-oxic zone almost solely consisted of *Halobacteria*, but the sublevel composition differed between the layers. Layer 1 was dominated by *Halobacteriaceae*, which were composed of OTUs exhibiting high similarities to *Halomarina*, *Halorussus*, and *Halorubrum*. The latter phylogenetic groups represented 69, 15, and 8% of all archaeal sequence in the layer, respectively. These genera comprise aerobic, halophilic and often phototrophic *Archaea*, which is in accordance with their localization [69]. The halobacterial genera detected in layer 1 disappeared in layers 2 and 3, which were dominated by uncultured members of the Deep Sea Hydrothermal Vent Group 6 (DHVEG-6). Sedimentation of these *Archaea* from lake water to the surface layer is possible and might explain the shift of the halobacterial subgroups. In the darker and more oxygen-depleted transition zone, *Halobacteria* strongly decrease in abundance and *Thermoplasmata* proliferate. *Archaea* of this group were affiliated to uncultured members of Marine Benthic Group D (MBG-D), DHVEG-1 and CCA47, which were all frequently found in marine deep anoxic environments such as sediments and hydrothermal vents [70]–[73]. A recent study showed that members of MBG-D are widely distributed in marine environments, preferentially in deeper anoxic sediments. At least some members of this group are involved in detrital protein degradation [74]. This is in accordance to their localization in the anoxic mat parts (layers 4 to 9) and might explain the in 2002 observed enhanced ammonia concentration of the mat pore water [19]. Therefore, this highly abundant group of the archaeal community might be involved in the recycling of detrital proteins from the EPS matrix. Uncultured members of the *Thaumarchaeota* increased in abundance from layers 6 to 9. In layers 7 and 8 *Thaumarchaeota* were the predominant archaeal group. Most of the corresponding 16S rRNA gene sequences were classified as members of MBG-B, which have been found in anoxic marine habitats such as Black Sea microbial mats and in the Guerrero Negro mat [10], [75]. Unfortunately, no cultivated representatives of this group exist to date, but it has been proposed that these organisms are sulfate-reducers [76]. Interestingly, members of *Methanomicrobia* and Group C3 (*Thaumarchaeota*) were only detected in significant abundances in layer 8. The class *Methanomicrobia* is capable of methanogenesis and therefore requires the strictly anoxic milieu of the deepest parts of the mat. This class was composed of two OTUs showing similarities to *Methanohalophilus* and *Methermicoccus.* The absence of methanogens in layer 9 might be due to groundwater-mediated influx of low oxygen levels. The 16S rRNA gene sequences affiliated to Group C3, which is a group of uncultured *Archaea* often found in low-temperature terrestrial and marine habitats [77], exhibited low similarities (<96%) to known 16S rRNA gene sequences. The trend of the archaeal group distribution along the depth gradient of the microbial mat is very similar to the reported distribution of two studies from Guerrero Negro [10], [13], pointing to a general occurrence of these archaeal groups in microbial mats. However, our current knowledge of *Archaea* in these habitats is limited, as cultured representatives are often lacking.

### Development of the Lake 21 Microbial Mat over the last Decade and Comparison with the Guerrero Negro Mat Community

The analysis of prokaryotic communities in this study revealed that almost all of the bacterial lineages, found in the previous 2002 mat sample of Lake 21 [19], were detected and are in accordance with the BHP analysis of the 2002 sample [22]. The latter study showed that cyanobacterial BHPs were located at the top part and proteobacterial BHPs in the lower part of the 2002 sampled mat [19], [22]. In addition, we found in 2011 representatives of different candidate phyla (BRC1, OP9, WS3, WS6, Hyd24-12, TA06), *Deferribacteres*, *Gemmatimonadetes*, and several other phyla and candidate divisions in low abundances (Figure 6 and 7; Figure S3 and S4). The additional bacterial groups detected in the 2011 sample may not have been detected in the prior study due to a smaller survey size. In addition, mat communities might have changed from 2002 to 2011 by overgrowth of the former 2002 mat community. A new mat community establishment is indicated, as the 2002 mat was thinner (1 cm at shoreline [19]) than that of 2011 (approximately 10 cm). In addition, 16S rRNA gene sequences from 2002 matched only sequences derived from deeper layers of the 2011 sampled mat. Correspondingly, the general lamination fabric of the mat changed, as more mat layers were observed in 2011 than in 2002. Microscopic observations showed that in 2002 frequently detected *Cyanobacteria* (*Microcoleus* and *Johannesbaptistia*) were in the present study only found in the lower layers of the mat or even trapped in gypsum crystals (Figure 2D–G). Thus, the bottom layers of the 2011 sampled mat resemble the 2002 mat layers. The observed mat structure in the present study might result from a collapse of “old” mat layers by microbial degradation of the EPS fabric and development of a new surface mat community.

**Figure 8:**
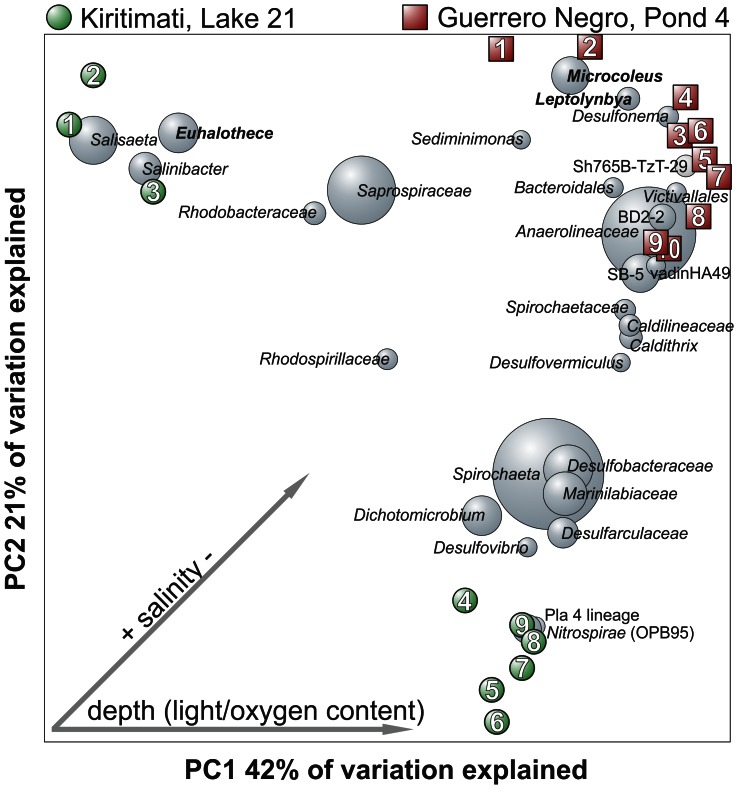
Comparison of Kiritimati and Guerrero Negro microbial mat layers by weighted principal coordinate analysis (PCoA). Numbers represent the layers (samples). The most abundant taxa (weighted average of all samples) are drawn in grey spheres. Sizes of the spheres are proportional to the mean relative abundance of the OTU across all samples. Potential environmental influences on community composition such as salinity, light and oxygen are illustrated by grey arrows.

Based on similarity searches, the overall prokaryotic community of the 2011 investigated Lake 21 mat showed more similarities to the Guerrero Negro microbial mat [9] than to the 2002 sampled Lake 21 mat [19]. Approximately 61% of the detected bacterial OTUs exhibited highest similarities to database entries derived from the Guerrero Negro microbial mat [9]. Generally, several significant similarities of phyla distribution and composition along the depth gradient were encountered, such as the almost ubiquitously distributed SRB and the high diversity of *Spirochaetes*. Comparison of the bacterial mat communities by PCoA revealed also several differences with respect to relative abundances (Figure 8). One example is the different cyanobacterial community in both microbial mat surfaces. *Euhalothece* dominated in Lake 21 mat, whereas *Microcoleus* and *Leptolyngbya* were most abundant in the Guerrero Negro mat. Another example is the abundance of *Nitrospirae* (OPB95), which occurred in high abundance in the anoxic zone (layer 9) of Lake 21, but were almost absent in the Guerrero Negro mat. Reasons for differences in community structures of Lake 21 and Guerrero Negro mat as well as the higher diversity of the Guerrero Negro bacterial community might be related to different salinities (170‰ Lake 21 and 80‰ Guerrero Negro). It is indicated that the filtering effect of higher salinities also affects the remaining bacterial and archaeal community structures towards presence of salt-resistant organisms [46].

### Microbialite Formation in Lake 21 Microbial Mat

Microbialite formation is an active process in the investigated Lake 21 mat (Figure 2C). The mat is characterized by different types of mineral precipitates: the surface is covered with gypsum crystals and to a lower amount with aragonite crystals, followed by small aragonite spherulites in layers 4, 6, and 7, large gypsum crystals in layer 5, and aragonitic, reticulate microbialites at the bottom of the mat, which already begin to form in the last two layers.

In general, control of microbialite formation is given at three different levels: (i) the macroenvironmental mineral saturation states, (ii) the metabolic activity of microbes affecting ion activities within the mats, and (iii) extracellular polymeric substances affecting mineral nucleation within the mats (for review see, e.g. [6], [19], [78]–[82]).

In Kiritimati Lake 21, CaCO_3_ mineral supersaturation is high, and the photosynthetic *Cyanobacteria* in the top layers further increase the Ca^2+^ × CO_3_^2−^ ion activity product significantly within the mat. However, microbialite formation largely takes place in deeper mat parts, while the photosynthetic top layers of the mats show only minor precipitates such as scattered aragonite spherulites [19]. This observation has been explained by a strong inhibition of precipitation in the top mat layers, and successive heterotrophic degradation of inhibiting exopolymers with depth [19].

**Figure 9:**
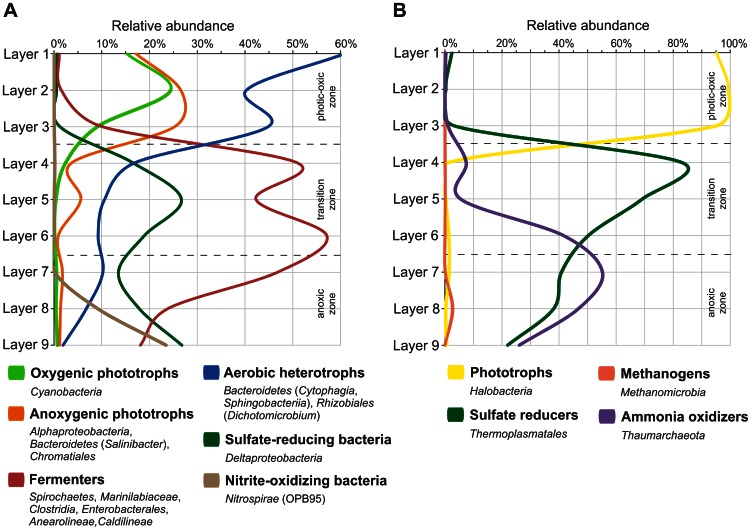
Spatial distribution and relative abundance of bacterial (A) and archaeal (B) functional groups.

The present data now substantiate the abundance of fermenting bacteria such as *Spirochaetes, Marinilabiaceae, Clostridia, Anaerolineae* and *Caldilineae*, which potentially degrade exopolymers in the transitional zone and anoxic zone. Besides of breakdown of nucleation inhibition, a secondary release of Ca^2+^ from the degraded exopolymers may further contribute to microbialite formation [79], [81]. Sulfate-reducing bacteria were almost absent in oxic top layers (Figure 9), contrary to reports from other microbialite forming settings (e.g., [55]). Instead, the distribution pattern of SRB rather matches the traditional view [83], i.e. high abundances in the transition and anoxic zones. While the metabolic effect of SRB on the CaCO_3_ mineral saturation is arguable, because pH and CaCO_3_ saturation states decrease in these zones [19], some of them may degrade exopolymers too [55], thereby contributing to the breakdown of nucleation inhibition. In turn, methanogens (*Methanomicrobia*) were only detected in low abundances and restricted to mat layer 8 - contrary to previous assumptions [19].

A complete decomposition of the mat exopolymer matrix is finally achieved at the mat bottom, where *Nitrospirae* and *Thaumarchaeota* are strikingly abundant (Figure 9). While *Nitrospirae* comprise nitrite-oxidizing as well as sulfate-reducing members, *Thaumarchaeota* are largely considered as ammonia oxidizers [84]. Indeed, high ammonium concentrations in the anoxic pore water of the microbialites [19] apparently foster the growth of these microbial groups at the mat bottom.

### Conclusions

Microbial mats act as self-controlling ecosystems by (re-)cycling the essential elements of life. The microbial mats from the Kiritimati lakes are excellent study objects to analyze the high complexity of microbial mat communities due to well-laminated mat layers, which allow high resolution spatial profiling. In addition, mineral precipitation under extreme environmental conditions such as high UV radiation and salinity can be studied. In accordance with the high oxygen level and light intensity, the prokaryotic communities in the surface layers of the Lake 21 mat are mainly composed of halophile oxygenic and anoxygenic phototrophs, and aerobic heterotrophs. In the transition zone, SRB, fermenting bacteria, and potential sulfate-reducing *Archaea* proliferate, while the abundance of phototrophic organisms declines. In the anoxic zone also SRB (with a different sublevel composition) and fermenters build the major part of the bacterial community. An involvement of both in microbialite formation is likely. Ammonia-oxidizing *Archaea* and low numbers of methanogens were also present in the deepest mat layers (Figure 9). In general, heterotrophic prokaryotes dominate the whole mat, as they nourish on organic matter supplied by phototrophic organisms from the photic-oxic zone. In addition, the diversity of bacterial and archaeal communities increased with depth. This result can be generalized for hypersaline microbial mats, as other DNA-based studies of microbial mats encountered a similar trend. The phylogenetic analyses of the mat also illustrates the gap of knowledge with respect to microbes inhabiting these ecosystems, as most of the detected 16S rRNA gene sequences showed low or no significant similarities to known and characterized microbes. Approximately 27% of the bacterial and 61% of the archaeal OTUs showed 16S rRNA gene sequence identities of lower than 95% including OTUs with low coverage values (<95%) to database entries, indicating at least the presence of novel species. In addition, most of these OTUs exhibited highest similarities to 16S rRNA gene sequences from uncultured and uncharacterized organisms. Thus, assessment of ecosystem functions of these microbes is difficult, as cultivated representatives are absent. To shed light on the key metabolic traits of the unknown taxa functional profiling of the microbial mat layers by direct DNA sequencing complemented with isolation and analysis of individual organisms will be performed in further studies. This would also allow linking phylogenetic composition and functional repertoire as well as unraveling lithification processes in these unique and robust ecosystems.

## Supporting Information

Figure S1 The daily number of hourly observed precipitation reports during 2010 and 2011.
(PDF)

Figure S2 Primer coverage of bacterial and archaeal phyla.
(PDF)

Figure S3 Relative abundances of rare bacterial phylogenetic groups.
(PDF)

Figure S4 Relative abundances of rare archaeal phylogenetic groups.
(PDF)

Table S1 Hydrochemical data of sea water and Lake 21 waters, Kiritimati atoll.
(PDF)

Table S2 Data processing of 16S rRNA gene sequences.
(PDF)

Table S3 Hydrochemical data of Lake 21 water and pore water of microbial mat layers.
(PDF)
