# Supplementary material for: Phylogenetic Analysis of a Microbialite-Forming Microbial Mat from a Hypersaline Lake of the Kiritimati Atoll, Central Pacific
Source: PLoS One. 2013 Jun 10;8(6):e66662. doi: 10.1371/journal.pone.0066662 (PMC3677903; doi:10.1371/journal.pone.0066662)
Supplement: Figure S1 — The daily number of hourly observed precipitation reports during 2010 and 2011. (PDF) [file pone.0066662.s001.pdf]

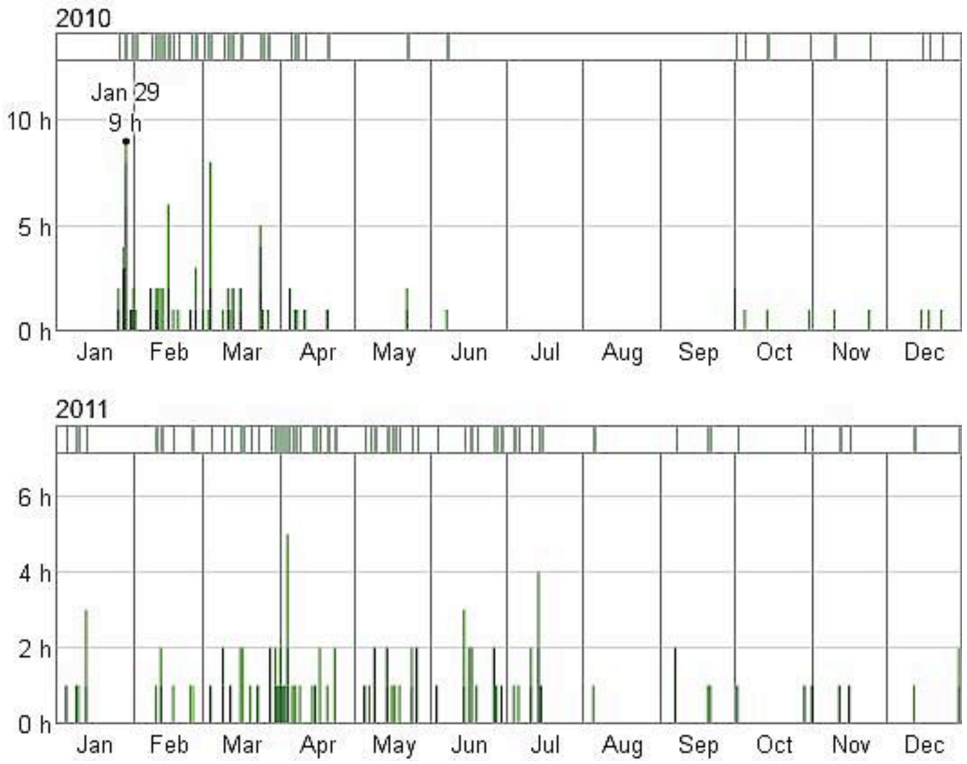

Figure S1. The daily number of hourly observed precipitation reports during 2010 and 2011. Color coded according to precipitation type and stacked in order of severity. From the bottom up, the categories are heavy, moderate, and light rain (dark to light green); and drizzle (lightest green). The faint shaded areas indicate climate normals. The bar at the top of the graph is green if any precipitation was observed that day and white otherwise (http://weatherspark.com).
