# Supplementary material for: Phylogenetic Analysis of a Microbialite-Forming Microbial Mat from a Hypersaline Lake of the Kiritimati Atoll, Central Pacific
Source: PLoS One. 2013 Jun 10;8(6):e66662. doi: 10.1371/journal.pone.0066662 (PMC3677903; doi:10.1371/journal.pone.0066662)
Supplement: Figure S2 — Primer coverage of bacterial and archaeal phyla. (PDF) [file pone.0066662.s002.pdf]

(A) V3for_B

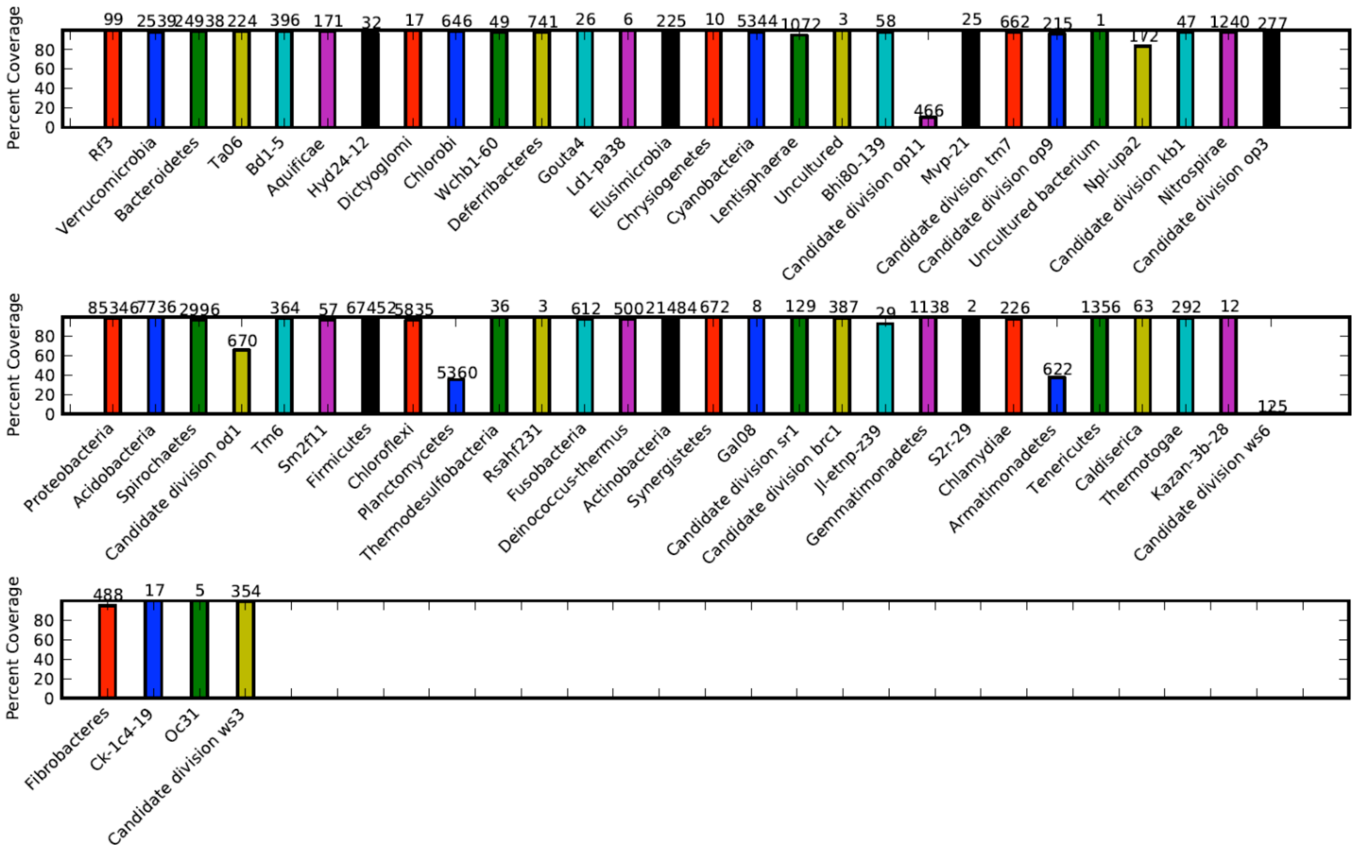
Percent Coverage
0
20
40
60
80
99
2539
24938
224
396
171
32
17
646
49
741
26
6
225
10
5344
1072
3
58
466
25
662
215
1
172
47
1240
277
Rf3
Verrucomicrobia
Bacteroidetes
Ta06
Bd1-5
Aquificae
Hyd24-12
Dictyoglomi
Chlorobi
Wchb1-60
Deferribacteres
Gouta4
Ld1-pa38
Elusimicrobia
Chrysiogenetes
Cyanobacteria
Lentisphaerae
Uncultured
Bhi80-139
Candidate division op11
Mvp-21
Candidate division tm7
Candidate division op9
Uncultured bacterium
Npl-upa2
Candidate division kb1
Nitrospirae
Candidate division op3
85346
7736
2996
670
364
57
67452
5835
5360
36
3
612
500
21484
672
8
129
387
29
1138
2
226
622
1356
63
292
12
125
Proteobacteria
Acidobacteria
Spirochaetes
Candidate division od1
Tm6
Sm2f11
Firmicutes
Chloroflexi
Planctomycetes
Thermodesulfobacteria
Rsahf231
Fusobacteria
Deinococcus-thermus
Actinobacteria
Synergistetes
Gal08
Candidate division sr1
Candidate division brc1
Jl-etnp-z39
Gemmatimonadetes
S2r-29
Chlamydiae
Armatimonadetes
Tenericutes
Caldiserica
Thermotogae
Kazan-3b-28
Candidate division ws6
488
17
5
354
Fibrobacteres
Ck-1c4-19
Oc31
Candidate division ws3

(B) V5rev_B

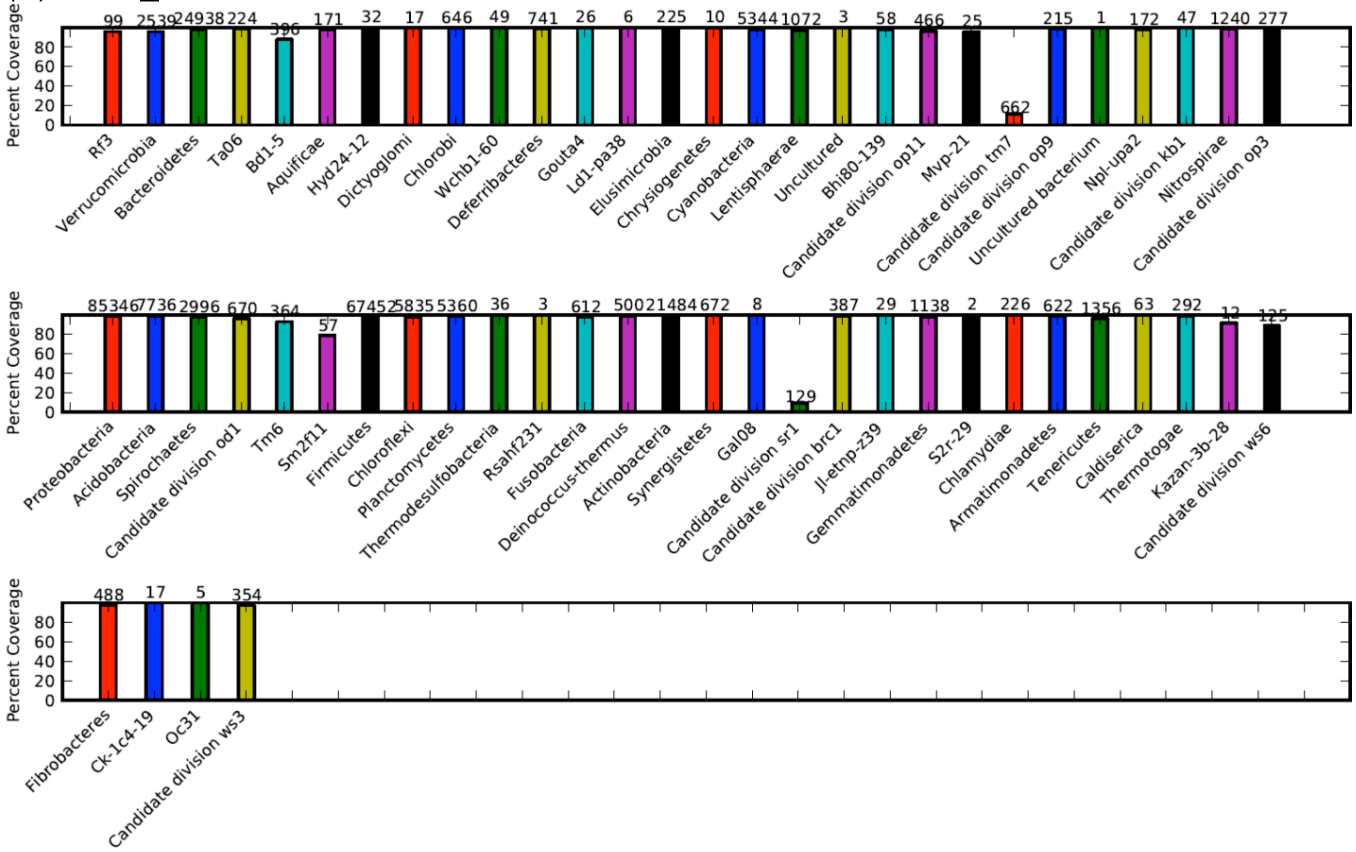

(C) V3for_A

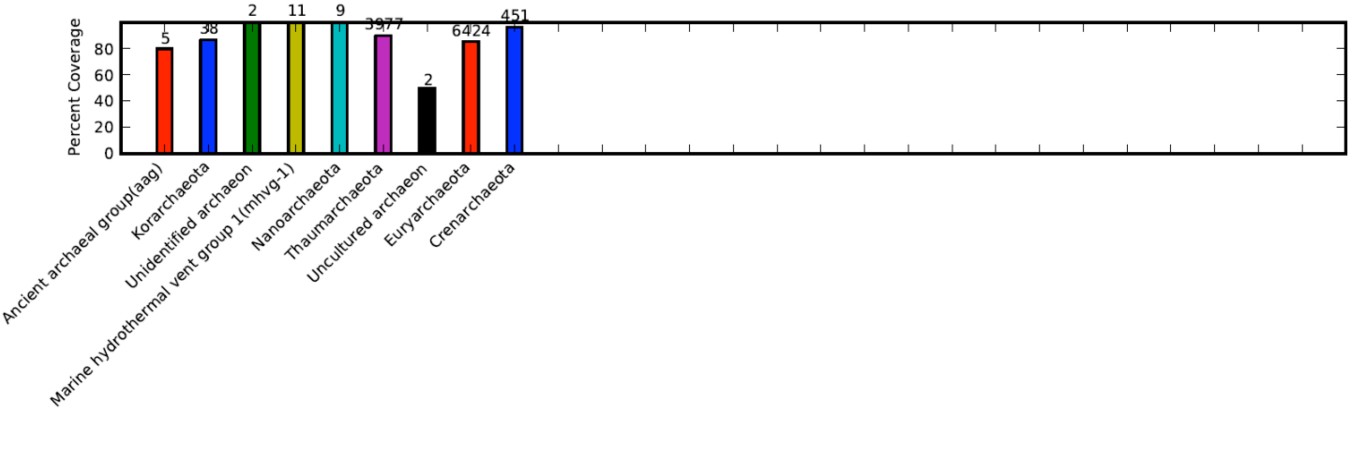

(D) V5rev_A

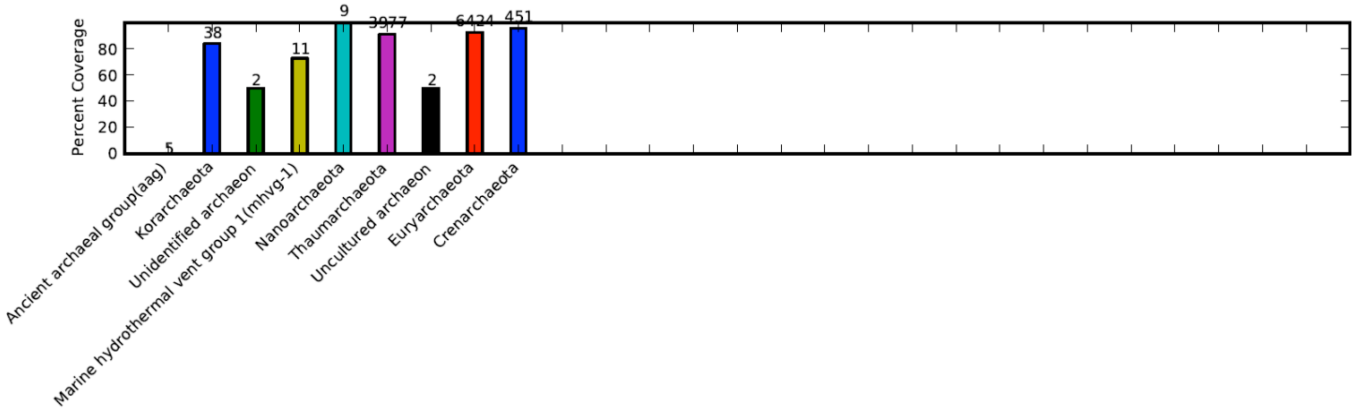

Figure S2. Primer coverage of bacterial and archaeal phyla. Coverage of bacterial phyla of forward (A) and reverse (B) primers and archaeal phyla of forward (C) and reverse (D) primers were determined with PrimerProspector [1]. Colored bars represent percent coverage of the primers and numbers above bars depict total sequences within the SILVA SSU database (release 111 Ref NR) for each phylum [2].

References

1. Walters WA, Caporaso JG, Lauber CL, Berg-Lyons D, Fierer N, et al. (2011) PrimerProspector: de novo design and taxonomic analysis of barcoded polymerase chain reaction primers. Bioinformatics 27: 1159-1161.

2. Pruesse E, Quast C, Knittel K, Fuchs BM, Ludwig W, et al. (2007) SILVA: a comprehensive online resource for quality checked and aligned ribosomal RNA sequence data compatible with ARB. Nucleic Acids Res 35: 7188-7196.
