# Supplementary material for: Phylogenetic Analysis of a Microbialite-Forming Microbial Mat from a Hypersaline Lake of the Kiritimati Atoll, Central Pacific
Source: PLoS One. 2013 Jun 10;8(6):e66662. doi: 10.1371/journal.pone.0066662 (PMC3677903; doi:10.1371/journal.pone.0066662)
Supplement: Figure S3 — Relative abundances of rare bacterial phylogenetic groups. (PDF) [file pone.0066662.s003.pdf]

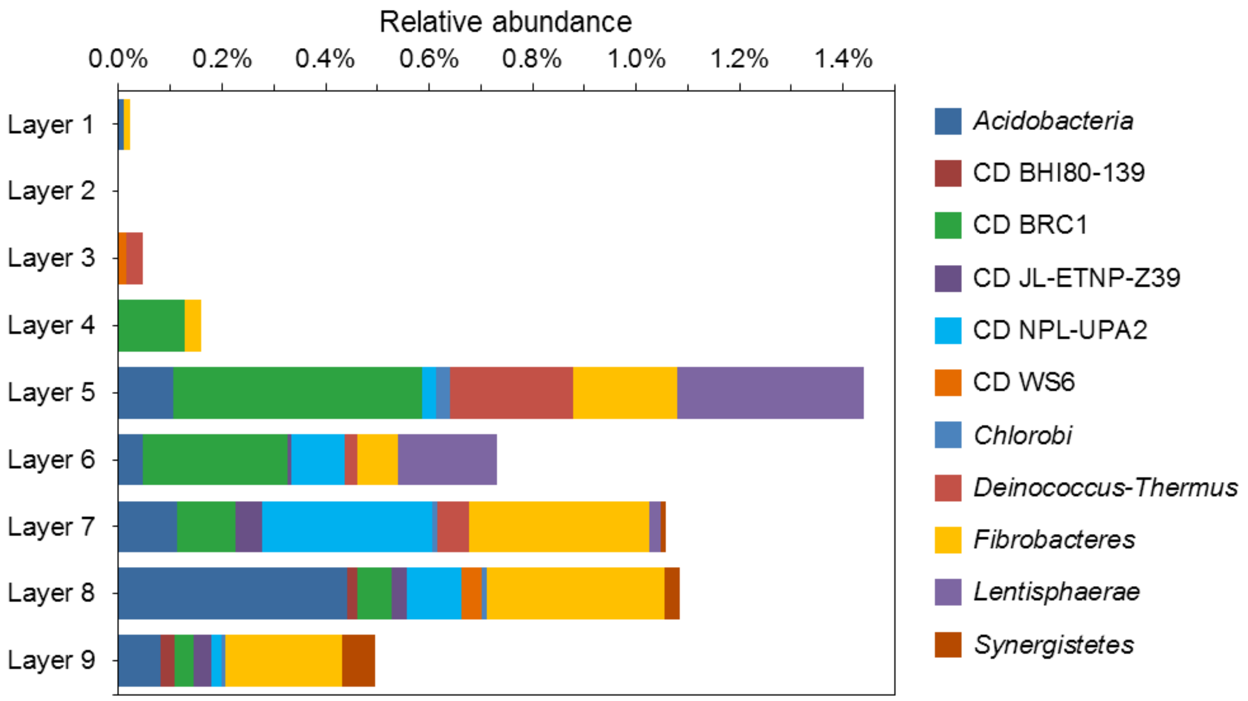

Figure S3. Relative abundances of rare bacterial phylogenetic groups. The artificial group "Other" (Figure 6) includes rare (<0.5%) bacterial phyla and candidate divisions (CD).
