# Supplementary material for: Phylogenetic Analysis of a Microbialite-Forming Microbial Mat from a Hypersaline Lake of the Kiritimati Atoll, Central Pacific
Source: PLoS One. 2013 Jun 10;8(6):e66662. doi: 10.1371/journal.pone.0066662 (PMC3677903; doi:10.1371/journal.pone.0066662)
Supplement: Table S1 — Hydrochemical data of sea water and Lake 21 waters, Kiritimati atoll. (PDF) [file pone.0066662.s005.pdf]

Table S1. Hydrochemical data of sea water and Lake 21 waters, Kiritimati atoll.

**Sample**	**Sampling depth [m]**	**Date**	**T [°C]**	**EC 25°C (mS cm^{-1})**	**Salinity (‰)**	**Eh (mV)**	**pH**
Seawater (Captain Cook Hotel)	0.1	19.03.2011	28.4	53.0	34.7	n.d.	8.36
Lake 21 (SE-side 4 m from shore)	0.1	13.03.2011	31.7	189.8	171.8	91	7.95
Lake 21 (seepage-affected lake bottom)	1.0	15.03.2011	28.7	124.8	94.4	n.d.	8.03
Lake 21 (surface, 2002)	0.1	04.09.2002	32.5	140.1	112.9	263	8.37

**Sample**	**O_2**	**Ca^{2+}**	**Mg^{2+}**	**Na^+**	**K^+ (mmol L^{-1})**	**Sr^{2+}**	**Cl^-**	**SO_4^{2-}**	**TA meq L^{-1}**
Seawater (Captain Cook Hotel)	0.198	10.4	54	474	10.3	0.093	552	28.6	2.31
Lake 21 (SE-side 4 m from shore)	0.079	29.3	299	2633	57.1	0.426	3054	143.3	4.84
Lake 21 (seepage-affected lake bottom)	n.d.	19.1	155	1363	29.7	0.232	1588	74.7	3.44
Lake 21 (surface, 2002)	n.d.	38.0	197	1643	34.7	0.312	1841	113.6	4.24

**Sample**	**Si**	**Fe (µmol L^{-1})**	**NH^{4+}**	**PO_4^{3-}**	**PCO_2 (µatm)**	**$SI_{Calcite}$[a]**	**$SI_{Aragonitea}$ (log IAP/KT)**	**SI_{Gypsum}[a]**
Seawater (Captain Cook Hotel)	3.6	1.10	n.d.	0.44	269	0.86	0.72	-0.66
Lake 21 (SE-side 4 m from shore)	54.6	1.35	0.086	0.02	871	1.26	1.12	0.13
Lake 21 (seepage-affected lake bottom)	n.d.	0.23	n.d.	n.d.	676	0.92	0.78	-0.26
Lake 21 (surface, 2002)	31.1	1.97	2.25	0.46	224	1.44	1.30	0.13

[a]Saturation index $SI_{Calcite}$ = log Ω_{Calcit}e = log [ion activity product (Ca^{2+}) × (CO_3^{2-}) / solubility product $K_{calcite}$]
n.d., not determined
