# Supplementary material for: Phylogenetic Analysis of a Microbialite-Forming Microbial Mat from a Hypersaline Lake of the Kiritimati Atoll, Central Pacific
Source: PLoS One. 2013 Jun 10;8(6):e66662. doi: 10.1371/journal.pone.0066662 (PMC3677903; doi:10.1371/journal.pone.0066662)
Supplement: Table S2 — Data processing of 16S rRNA gene sequences. (PDF) [file pone.0066662.s006.pdf]

Table S2. Data processing of 16S rRNA gene sequences**.**

Sample	No. of sequences												
	**Raw**	**<250 bp**	**With ambiguous base pairs**	**Quality score <25**	**>8 homo-polymers**	**>2 mismatches in forward primer**	**After quality filtering**	**Potential chimeras (UCHIME *de novo*)**	**Potential chimeras (UCHIME reference mode)**	**In OTU table**	**Singletons**	**Extrinsic domain and chloroplast OTUs**	**Final OTU table**
Bacteria													
Layer 1	13,523	2,997	551	89	0	106	9,780	4	0	9,776	315	16	9,445
Layer 2	9,965	2,809	424	35	0	28	6,669	3	0	6,666	19	1	6,646
Layer 3	10,604	3,297	451	27	0	38	6,791	374	9	6,408	93	0	6,315
Layer 4	10,418	3,080	741	16	0	36	6,545	81	1	6,463	184	0	6,279
Layer 5	11,200	2,705	773	51	0	46	7,625	53	4	7,568	63	0	7,505
Layer 6	17,981	4,283	835	87	2	106	12,668	6	0	12,662	82	0	12,580
Layer 7	15,064	4,086	906	84	3	64	9,921	6	1	9,914	173	0	9,741
Layer 8	16,653	4,268	1,195	106	3	55	11,026	5	2	11,019	602	0	10,417
Layer 9	17,308	4,697	948	133	1	41	11,488	1	0	11,487	370	0	11,117
Archaea													
Layer 1	6,867	998	458	937	9	1,323	3,142	0	0	3,142	35	0	3,107
Layer 2	4,980	710	149	33	0	274	3,814	7	0	3,807	2	0	3,805
Layer 3	4,585	646	223	50	0	219	3,447	12	0	3,435	17	0	3,418
Layer 4	5,101	607	334	43	0	376	3,741	4	0	3,737	213	0	3,524
Layer 5	22,937	5,088	711	3,071	1	2,519	11,547	852	0	10,695	31	1	10,663
Layer 6	13,985	2,345	335	2,060	4	1,956	7,285	28	0	7,257	22	0	7,235
Layer 7	5,933	959	160	819	2	824	3,169	73	0	3,096	16	0	3,080
Layer 8	4,859	745	166	758	0	453	2,737	38	0	2,699	155	0	2,544
Layer 9	5,061	954	137	876	0	617	2,477	54	0	2,423	69	1	2,353
