# Supplementary material for: Phylogenetic Analysis of a Microbialite-Forming Microbial Mat from a Hypersaline Lake of the Kiritimati Atoll, Central Pacific
Source: PLoS One. 2013 Jun 10;8(6):e66662. doi: 10.1371/journal.pone.0066662 (PMC3677903; doi:10.1371/journal.pone.0066662)
Supplement: Table S3 — Hydrochemical data of Lake 21 water and pore water of microbial mat layers. (PDF) [file pone.0066662.s007.pdf]

Table S3. Hydrochemical data of Lake 21 water and pore water of microbial mat layers.

Sample	pH	Temperature (°C)	Redox potential (mV)
Lake water	7.948	31.7	91.4
Layer 1[a]	7.708	32	-43
Layer 2[a]	7.708	32	-43
Layer 3[a]	7.708	32	-43
Layer 4	6.716	32	-123
Layer 5	6.670	32	-133
Layer 6	6.664	32	-142
Layer 7[a]	6.661	32	-135
Layer 8[a]	6.661	32	-135
Layer 9	Not determined	Not determined	Not determined

[a]values derive from pooled porewater
